# Reconstruction of fluorescence molecular tomography with a cosinoidal level set method

**DOI:** 10.1186/s12938-017-0377-0

**Published:** 2017-06-27

**Authors:** Xuanxuan Zhang, Xu Cao, Shouping Zhu

**Affiliations:** Engineering Research Center of Molecular and Neuro Imaging of the Ministry of Education & School of Life Science and Technology, Xidian University, Xi’an, 710071 Shaanxi China

**Keywords:** Fluorescence molecular tomography, Level set method, Levenberg–Marquardt method

## Abstract

**Background:**

Implicit shape-based reconstruction method in fluorescence molecular tomography (FMT) is capable of achieving higher image clarity than image-based reconstruction method. However, the implicit shape method suffers from a low convergence speed and performs unstably due to the utilization of gradient-based optimization methods. Moreover, the implicit shape method requires *priori* information about the number of targets.

**Methods:**

A shape-based reconstruction scheme of FMT with a cosinoidal level set method is proposed in this paper. The Heaviside function in the classical implicit shape method is replaced with a cosine function, and then the reconstruction can be accomplished with the Levenberg–Marquardt method rather than gradient-based methods. As a result, the *priori* information about the number of targets is not required anymore and the choice of step length is avoided.

**Results:**

Numerical simulations and phantom experiments were carried out to validate the proposed method. Results of the proposed method show higher contrast to noise ratios and Pearson correlations than the implicit shape method and image-based reconstruction method. Moreover, the number of iterations required in the proposed method is much less than the implicit shape method.

**Conclusions:**

The proposed method performs more stably, provides a faster convergence speed than the implicit shape method, and achieves higher image clarity than the image-based reconstruction method.

## Background

Thanks to the ever-increasing fluorescent probes, proteins, and dyes, quantities of in vivo biomedical researches at cellular and subcellular levels are achieved noninvasively such as protein–protein interactions, protein function and gene expression [1–3]. With the advancement of fluorescent markers, a number of fluorescent imaging techniques now are available to visualize them at either microscopic [4–7] or macroscopic scale [8–10]. Fluorescence molecular tomography (FMT) [10–12] is a typical macroscopic fluorescent imaging technique that noninvasively reveals the distributions of fluorescent markers inside the bodies of small animals through fluorescent measurements at the surfaces of bodies, which has been applied for drug discovery [13, 14] and oncology [15, 16].

The concept of FMT can be summarized as: the excitation and fluorescent light propagations in tissues are described with a certain mathematical model firstly, and then, a reconstruction scheme is conceived based on the minimization of the differences between fluorescent measurements and the corresponding predicted ones through the model. The two processes are generalized as two problems: forward and inverse problems [17]. Commonly, diffusion equation, an approximation of radiative transfer equation, is used to model the light propagations in tissues in forward problem [18]. As a partial differential equation, diffusion equation is usually solved with numerical methods such as finite element method (FEM) [19]. On the other hand, because the fluorescent measurements used in reconstruction are only obtained at the surfaces, inverse problem is ill-posed, which makes reconstruction results sensitive to measurement noise and numerical errors. To overcome the ill-posedness, inverse problem is treated as an optimization problem with regularizations and a list of numerical methods can be applied to solve it such as Newton method [17] and conjugate gradient method [18]. In reconstruction, the value of fluorescent yield of each node, pixel, or voxel is recovered from a set of fluorescent measurements obtained from different projection angles. However, the high ill-posedness of inverse problem and the utilization of regularization result in a poor spatial resolution that the boundaries of reconstructed objects are blurred [20].

The blurry images inhibit the applications of FMT in some occasions that need explicit boundaries [20]. To deal with these cases, shape-based reconstruction methods [20–28] are developed, which parameterize the shapes of reconstructed objects and recover these shape parameters instead of the values of fluorescent yield at each node, pixel, or voxel in classical image-based reconstruction schemes. In general, shape-based reconstruction methods can be classified into two types: implicit [20–24] and explicit shape methods [25–28]. Explicit shape method describes the boundaries of reconstructed objects with a spherical harmonics expansion and the expansion coefficients are reconstructed to construct the images. Implicit shape method defines the shapes of reconstructed objects with a level set function, which is updated during reconstruction iterations to recover the boundaries. Both of the two types are capable of recovering arbitrary shapes theoretically, but the complexity of the shapes defined by spherical harmonics expansion is restricted by the maximum degree of spherical harmonics which is limited and determined manually in practical applications [25].

Shape-based reconstruction methods are capable of achieving higher image clarity than the image-based reconstruction methods. However, *priori* information about the number of reconstructed objects is essential for both of the two types of shape-based reconstruction methods. For the spherical harmonics expansion, a set of expansion coefficients can only describe a single object. Thus, multi-object reconstruction needs more than one set of expansion coefficients and each one needs to be initialized at the beginning of reconstruction [25]. In parallel, the definition of multiple objects needs multiple levels of a single level set function or more than one level set function and the initializations of both need to know the number of objects [29, 30]. Moreover, in the implicit shape method, reconstruction is commonly accomplished through an artificial time evolution approach which utilizes gradient-based optimization methods to update the level set function such as the gradient descent method [20–24]. The gradient-based optimization methods are first order methods which suffer from a low convergence speed and sometimes converge to a local minimum [31]. In addition, the choice of step length is also an intractable problem, which controls the convergence speed and the calculation accuracy.

Second order methods, e.g. Newton-type methods, converge quadratically, which benefits from the utilization of the second derivative of object function. Compared to first order methods, second order methods converge more quickly and perform more stably [31]. However, the implicit shape method is failure to take advantage of second order methods due to the non-differentiability of the derivative of the Heaviside function. In this paper, a shape-based reconstruction scheme of FMT with cosinoidal level set method is conceived to take use of Newton-type method. This reconstruction method replaces the Heaviside function with a cosine function in the classical implicit shape method so as to obtain the second derivative of object function. Simulation and phantom studies are implemented to validate the performance of the proposed method.

## Methods

The lights with wavelength between 700 and 900 nm are highly scattered and lowly absorbed in tissues, which are called diffuse lights commonly. Diffuse lights are appropriate for macroscopic imaging because of the high tissue penetration. Due to the high scattering property, diffusion equation is usually used to describe the propagations of diffuse light [18]. Because the generation of fluorescence consists of two processes (excitation and emission), a couple of diffusion equations are commonly used to describe the propagations of the excitation light and fluorescence, which are converted into linear equations through FEM as follows [32, 33]:

1 $$\left\{ {\begin{array}{*{20}c} {KU_{x} = Q} \\ {KU_{m} = FX} \\ \end{array} } \right.$$

where *U* is the photon density and *Q* is the source term. The subscripts *x* and *m* are used to discriminate between the excitation and the emission. *U* and *Q* are column vectors with *N*_*n*_ elements. *N*_*n*_ denotes the number of nodes used in FEM. *X* is the vector of fluorescent yield with the same length with *U* and *Q*, which is the unknown vector to be reconstructed. *K* is the stiffness matrix with *N*_*n*_ × *N*_*n*_ elements. *F* is a matrix with the same size with *K*. The elements of *K*, *F*, and *Q* are given by:

2 $$K_{ij} = \int\limits_{\varOmega } {(D\nabla \upsilon_{i} \cdot \nabla \upsilon_{j} + \mu_{a} \upsilon_{i} \upsilon_{j} )dr^{n} } + \frac{1}{2q}\int\limits_{\partial \varOmega } {\upsilon_{i} \upsilon_{j} dr^{n - 1} }$$

3 $$Q_{i} = \int\limits_{\varOmega } {Q(r)\upsilon_{i} dr^{n} }$$

4 $$F_{ij} = \int\limits_{\varOmega } {U_{x} (r)\upsilon_{i} \upsilon_{j} dr^{n} }$$

where *μ*_*a*_ is the absorption coefficient, *D* is the diffusion coefficient, *q* is a term related to the optical reflective index mismatch at the boundary, *r* is the position, *υ* represents the shape function, and the subscripts *i* and *j* denote the indices of column and row, respectively.

In the implicit shape method, the level set function is introduced to express the distribution of the unknown parameter as follows [21]:

5 $$x(r) = \left\{ {\begin{array}{*{20}l} {\begin{array}{*{20}l} {x_{f} } & {\psi (r) \le 0} \\ \end{array} } \\ {\begin{array}{*{20}l} {x_{b} } & {\psi (r) > 0} \\ \end{array} } \\ \end{array} } \right.$$

where *x* denotes the fluorescent yield and *ψ* represents the level set function. The subscripts *f* and *b* denote the regions of fluorescent targets and background, respectively. To obtain the gradient used in reconstruction, the Heaviside function *H*(*ψ*) is used to express *x*(*r*) in the classical implicit shape method [21]:

6 $$x(\psi ) = x_{b} H(\psi ) + x_{f} [1 - H(\psi )]$$

Equation (6) is capable of describing the distribution of unknown parameter with the level set function. However, this equation is only appropriate for the cases with a single object or multiple objects with the same fluorescent yield. For the cases with multiple objects with different fluorescent yields, a level set function with multiple levels or more than one level set function should be used to express the distribution of fluorescent yield and the number of the levels or level set functions is determined by the number of objects [29, 30]. Consequently, for the classical implicit shape method, *priori* information about the number of objects is required to initialize the configuration of level set function and fluorescent yield. Moreover, the derivative of the Heaviside function is the Dirac function, which cannot be differentiated further. This leads to the inability of the applications of second order methods in reconstruction. To solve these problems, the following equation is used to describe the distribution of fluorescent yield:

7 $$x(\psi ) = \frac{1}{2}[1 + \cos (\pi \psi )]x_{b} + \frac{1}{2}[1 - \cos (\pi \psi )]x_{f}$$

Equation (7) replaces the pair of Heaviside functions with a pair of cosine function. When the level set function *ψ* varies within [0,1], the value of fluorescent yield *x*(*ψ*) varies between *x*_*b*_ and *x*_*f*_. Compared to Eq. (6), the advantage of Eq. (7) is that the value of fluorescent yield is not restricted at only two values but varies between two values, i.e., Eq (7) is capable of the representation of multiple objects with different fluorescent yields. As a consequence, the number of objects is not required any more. In addition, the derivative of cosine function is sine function, which can be further differentiated. Therefore, second order methods can be applied.

From the second equation of Eq. (1), equation *U*_*m*_=*K* ^−1^ *FX*=*AX* can be obtained. In reconstruction, measurements acquired from different projection angles are corresponding to different fluorescent photon density *U*_*m*_ and matrix *A*. Extracting all the elements of *U*_*m*_ and rows of *A* according to the nodes on the surface for measurements and assembling them yields the vector of measurements *Y* and the Jacobian matrix *J* with respect to the fluorescent yield *x*. Then the following equation can express the relationship between the measurements *Y* and the fluorescent yield *X*:

8 $$Y = JX$$

To reconstruct the fluorescent yield *X* from the measurements *Y*, an object function is defined as follows:

9 $$\varGamma = \frac{1}{2}\left\| {JX - Y} \right\|_{2}^{2} = \frac{1}{2}\sum\limits_{i = 1}^{{N_{m} }} {\left(\sum\limits_{j = 1}^{{N_{n} }} {J_{ij} X_{j} } - Y_{i} \right)^{2} }$$

where *J*_*ij*_ denotes the elements of the matrix *J* at the *i*th row and *j*th column. *N*_*m*_ is the number of measurements.

The level set function *ψ* is discretized into a vector *Ψ* with the basis expansion of shape functions as follows:

10 $$\psi (r) = \sum\limits_{i = 1}^{{N_{n} }} {\varPsi_{i} \upsilon_{i} }$$

Then differentiating the object function *Γ* shown in Eq. (9) with respect to the level set function of a certain node *Ψ*_*k*_ yields:

11 $$\frac{\partial \varGamma }{{\partial \varPsi_{k} }} = \sum\limits_{i = 1}^{{N_{m} }} {\left( {\sum\limits_{j = 1}^{{N_{n} }} {J_{ij} X_{j} } - Y_{i} } \right)J_{ik} \frac{{\partial X_{k} }}{{\partial \varPsi_{k} }}}$$

From Eq. (7) the derivative of *X*_*k*_ with respect to *Ψ*_*k*_ can be obtained:

12 $$\frac{{\partial X_{k} }}{{\partial \varPsi_{k} }} = \frac{\pi }{2}(x_{f} - x_{b} )\sin (\pi \varPsi_{k} )$$

Assembling the derivatives of the object function with respect to the level set function for all the nodes yields:

13 $$\varGamma^{\prime} = \left[ {\begin{array}{*{20}c} {\frac{\partial \varGamma }{{\partial \varPsi_{1} }}} & \cdots & {\frac{\partial \varGamma }{{\partial \varPsi_{{N_{n} }} }}} \\ \end{array} } \right]^{T} = J_{\psi }^{T} (JX - Y)$$

14 $$J_{\psi ij} = \frac{\pi }{2}(x_{f} - x_{b} )J_{ij} \sin (\pi \varPsi_{j} )$$

where *J*_*ψ*_ is the Jacobian matrix with respect to the level set function *ψ*.

Further differentiating Eq. (13) with respect to the level set function yields the second derivative of the object function as follows:

15 $$\frac{{\partial ({{\partial \varGamma } \mathord{\left/ {\vphantom {{\partial \varGamma } {\partial \varPsi_{k} }}} \right. \kern-0pt} {\partial \varPsi_{k} }})}}{{\partial \varPsi_{l} }} = \sum\limits_{i = 1}^{{N_{m} }} {J_{\psi ik} J_{\psi il} } + \sum\limits_{i = 1}^{{N_{m} }} {\frac{{\partial J_{\psi ik} }}{{\partial \varPsi_{l} }}\left( {\sum\limits_{j = 1}^{{N_{n} }} {J_{ij} X_{j} - Y_{i} } } \right)}$$

16 $$\frac{{\partial J_{\psi ik} }}{{\partial \varPsi_{l} }} = \left\{ {\begin{array}{*{20}l} {0 \quad l \ne k} \hfill \\ {\frac{{\pi^{2} }}{2}(x_{f} - x_{b} )J_{ij} \cos (\pi \varPsi_{k} ) \quad l = k} \hfill \\ \end{array} } \right.$$

Assembling the second derivatives of the object function with respect to the level set function for all the nodes yields:

17 $$\varGamma^{\prime\prime} = \left[ {\begin{array}{*{20}c} {\frac{{\partial ({{\partial \varGamma } \mathord{\left/ {\vphantom {{\partial \varGamma } {\partial \varPsi_{1} )}}} \right. \kern-0pt} {\partial \varPsi_{1} )}}}}{{\partial \varPsi_{1} }}} & \cdots & {\frac{{\partial ({{\partial \varGamma } \mathord{\left/ {\vphantom {{\partial \varGamma } {\partial \varPsi_{{N_{n} }} )}}} \right. \kern-0pt} {\partial \varPsi_{{N_{n} }} )}}}}{{\partial \varPsi_{1} }}} \\ \vdots & \ddots & \vdots \\ {\frac{{\partial ({{\partial \varGamma } \mathord{\left/ {\vphantom {{\partial \varGamma } {\partial \varPsi_{1} )}}} \right. \kern-0pt} {\partial \varPsi_{1} )}}}}{{\partial \varPsi_{{N_{n} }} }}} & \cdots & {\frac{{\partial ({{\partial \varGamma } \mathord{\left/ {\vphantom {{\partial \varGamma } {\partial \varPsi_{{N_{n} }} )}}} \right. \kern-0pt} {\partial \varPsi_{{N_{n} }} )}}}}{{\partial \varPsi_{{N_{n} }} }}} \\ \end{array} } \right] = J_{\psi }^{T} J_{\psi } + H_{\psi } \left[ {\begin{array}{*{20}c} {JX - Y} & {} & {} \\ {} & \ddots & {} \\ {} & {} & {JX - Y} \\ \end{array} } \right]$$

where *H*_*ψ*_ is the Hessian matrix with respect to the level set function and can be expressed as:

18 $$H_{\psi } = \left[ {\begin{array}{*{20}c} {\frac{{\partial J_{\psi 11} }}{{\partial \varPsi_{1} }}} & \cdots & {\frac{{\partial J_{{\psi N_{m} 1}} }}{{\partial \varPsi_{1} }}} & {\frac{{\partial J_{\psi 12} }}{{\partial \varPsi_{1} }}} & \cdots & {\frac{{\partial J_{{\psi N_{m} 2}} }}{{\partial \varPsi_{1} }}} & \cdots & {\frac{{\partial J_{{\psi N_{m} N_{n} }} }}{{\partial \varPsi_{1} }}} \\ \vdots & \ddots & \vdots & \vdots & \ddots & \vdots & \ddots & \vdots \\ {\frac{{\partial J_{\psi 11} }}{{\partial \varPsi_{{N_{n} }} }}} & \cdots & {\frac{{\partial J_{{\psi N_{m} 1}} }}{{\partial \varPsi_{{N_{n} }} }}} & {\frac{{\partial J_{\psi 12} }}{{\partial \varPsi_{{N_{n} }} }}} & \cdots & {\frac{{\partial J_{{\psi N_{m} 2}} }}{{\partial \varPsi_{{N_{n} }} }}} & \cdots & {\frac{{\partial J_{{\psi N_{m} N_{n} }} }}{{\partial \varPsi_{{N_{n} }} }}} \\ \end{array} } \right]$$

Then Eqs. (13) and (17) are substituted into the Newton method ($x^{(n + 1)} = x^{(n)} - (\varGamma^{\prime\prime})^{ - 1} \varGamma^{\prime}$, where *x* denotes the unknown parameters and the superscript *n* denotes the index of iteration) [18] to obtain the iteration equation as follows:

19 $$\varPsi^{(n + 1)} = \varPsi^{(n)} - (J_{\psi }^{T} J_{\psi } + Hb)^{-1}J_{\psi }^{T} (JX - Y)$$

where *b* represents the matrix consisting of residual vectors on the right side of Eq. (17). To simplify the calculations and take advantage of regularization, the Levenberg–Marquardt (LM) method [18] is used to reconstruct the level set function instead of Eq. (19):

20 $$\varPsi^{(n + 1)} = \varPsi^{(n)} - (J_{\psi }^{T} J_{\psi } + \lambda I)^{-1}J_{\psi }^{T} (JX - Y)$$

where *I* denotes the identity matrix and *λ* is a regularization parameter. The LM method is a variation of the Newton method but more useful in practical applications, which ignores the Hessian matrix to reduce the computational requirements and introduces a regularization term to suppress the influence of noise. Compared with the original Newton method, the LM method provides a similar convergence speed but consumes less computational time and less storage space.

In parallel, the iteration equation for the fluorescent yields *x*_*b*_ and *x*_*f*_ can be acquired through the derivative of object function with respect to *x*_*b*_ and *x*_*f*_:

21 $$\left[ {\begin{array}{*{20}c} {x_{b} } \\ {x_{f} } \\ \end{array} } \right]^{(n + 1)} = \left[ {\begin{array}{*{20}c} {x_{b} } \\ {x_{f} } \\ \end{array} } \right]^{(n)} - (J_{x}^{T} J_{x} + \lambda I)^{-1}J_{x}^{T} (JX - Y)$$

22 $$J_{x} = \frac{1}{2}\left[ {\begin{array}{*{20}c} {J(1 + \cos (\pi \varPsi ))} & {J(1 - \cos (\pi \varPsi ))} \\ \end{array} } \right]$$

Equations (20) and (21) are used to reconstruct the level set function and fluorescent yields, respectively. During the reconstruction, the update of the level set function and fluorescent yields is carried out separately. Within each iteration, the level set function *Ψ* is updated through Eq. (20) firstly, and then the fluorescent yields *x*_*b*_ and *x*_*f*_ are updated through Eq. (21). After the update of the level set function and fluorescent yields, a restriction process is executed to ensure the level set function within [0,1]. When *ψ* < 0 the level set function is set as 0. When *ψ* > 1 the level set function is set as 1.

## Results and discussion

**Fig. 1:**
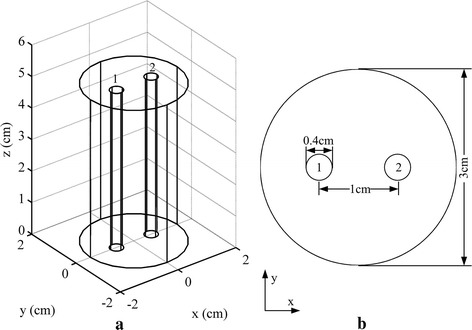
Geometry of the imaged object used in simulations and phantom experiments. **a** 3D view of the geometry of the cylindrical object. **b** Top view of the geometry

**Fig. 2:**
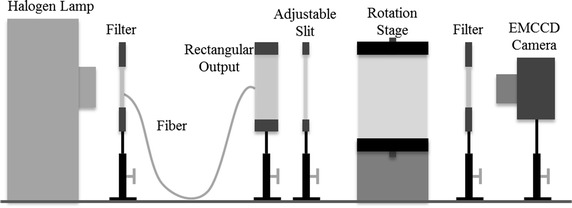
Schematic of the free-space FMT system

In order to validate the performance of the proposed method, numerical simulations and phantom experiments were carried out. The geometry of the imaged object used in the simulations and phantom experiments was a cylinder with a diameter of 3 cm and a height of 5 cm as shown in Fig. 1. Two tubes with a diameter of 0.4 cm and a height of 5 cm were inserted into the cylinder as the fluorescent targets. The distance between the centers of the two tubes was 1 cm. In the simulations, fluorescent measurements were generated through Eq. (1) and contaminated with 1% Gaussian noise. In the phantom experiments, a free-space FMT system [34] was used to acquire the fluorescent measurements. A schematic of the imaging system is shown in Fig. 2. A 250W Halogen lamp (7ILT250, 7-star, Beijing, China) was used as the excitation light source. A 775 ± 23 nm band pass filter (FF01-775/46-25, Semrock, Rochester, NY, USA) was placed toward the lamp and coupled with a special optical fiber. The output of the fiber was rectangular beam which was converted into line-shaped beam through an adjustable slit. The imaged object was placed on a rotation stage for full-angle projection measurements. An electron multiplying charge-coupled device (EMCCD) camera (iXon DU-897, Andor Technologies, Belfast, Northern Ireland) coupled with a Nikkor 60 mm f/2.8D lens (Nikon, Melville, NY, USA) and an 840 ± 6 nm bandpass filter (FF01-840/12-25, Semrock, Rochester, NY, USA) was implemented to capture the images. In both the simulations and phantom experiments, two different groups of measurements were obtained for the test of the cases with single or double targets. Tubes 1 and 2 were filled with 1.7 and 1.02 μmol/L indocyanine green (ICG) in the phantom experiments for the measurements of double targets, respectively, whereas only tube 1 was filled with 1.02 μmol/L ICG for those of single target. In addition, 1% intralipid with a reduced scattering coefficient of 10 cm^−1^ and an absorption coefficient of 0.02 cm^−1^ was used to fill the cylindrical object to simulate the tissues. Accordingly, the fluorescent yields of the two targets used in the simulations were set to 1 and 0.6, respectively, and the optical coefficients were set as the same with the phantom experiments. For each group of the measurements, a line source was utilized to illuminate the cylinder along z-axis and 36 fluorescent images of different projection angles were acquired.

In reconstruction, the distributions of fluorescent yield at the central slice z = 2.5 cm were recovered through the proposed method, the classical image-based reconstruction method as well as the implicit shape method. The LM method was implemented to accomplish the iteration in the proposed and image-based reconstruction method, while the gradient descent method based on the artificial time evolution approach [23] was used in the implicit shape method. A mesh with 1352 nodes and 2602 elements was used in the simulations while another one with 1473 nodes and 2800 elements was utilized for the phantom experiments. The reconstruction was terminated after 5 iterations for the LM method whereas 500 iterations were executed for the gradient descent method. The initial values of fluorescent yield used in the image-based reconstruction method were set to 0, while the initial values of level set function in the proposed and implicit shape method were set to 0.5 and 0.05, respectively. In addition, the reconstructions through the implicit shape method were implemented without the *priori* information about the number of targets, i.e. only a single level set function was used and the fluorescent yield was initialized with a background coefficient *x*_*b*_ and a single target coefficient *x*_*f*_.

**Fig. 3:**
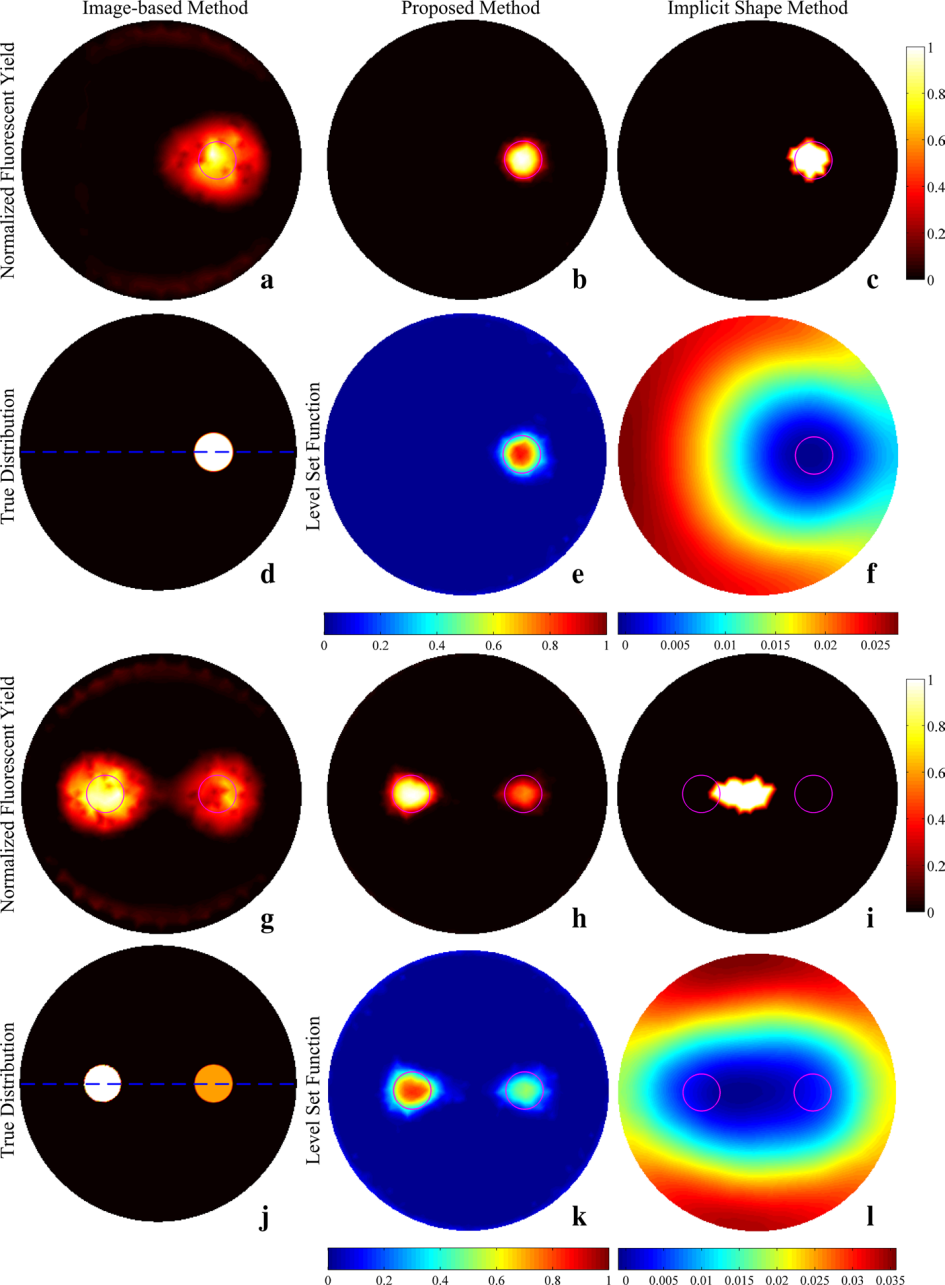
Reconstruction results of simulations. **a**–**c** Distributions of fluorescent yield reconstructed with the image-based method, the proposed method, and the implicit shape method for single target, respectively. **d** True distribution of fluorescent yield for single target. **e**, **f** Distributions of level set function reconstructed with the proposed method and the implicit shape method for single target, respectively. **g**–**l** Corresponding results for double targets. The reconstructions with the implicit shape method were implemented without the *priori* information about the number of targets

**Fig. 4:**
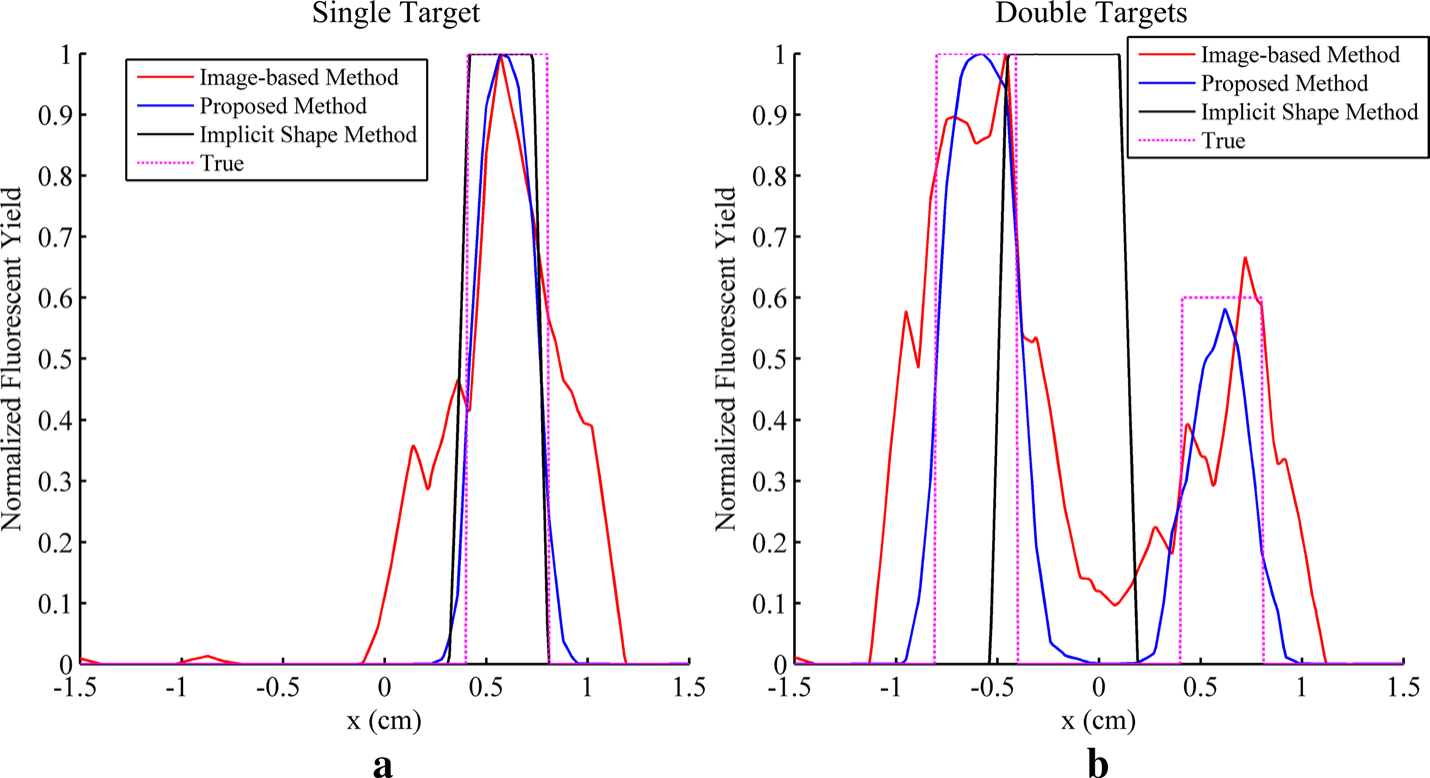
Profiles of normalized fluorescent yield along the blue dotted lines in Fig. 3d and j. **a** Result for Fig. 3d. **b** Result for Fig. 3j

**Fig. 5:**
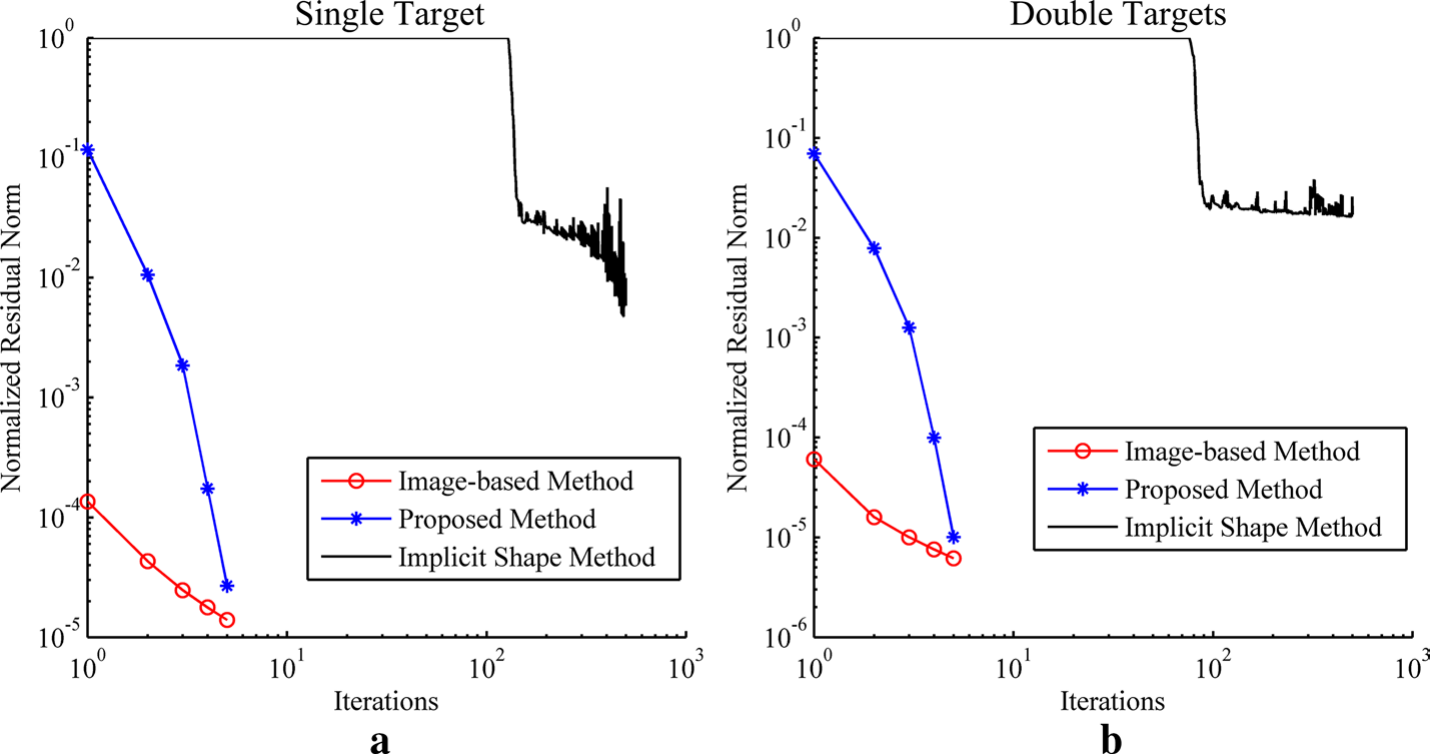
Residual norms as a function of iteration indices for simulation studies. **a** Result for single target. **b** Result for double targets

**Fig. 6:**
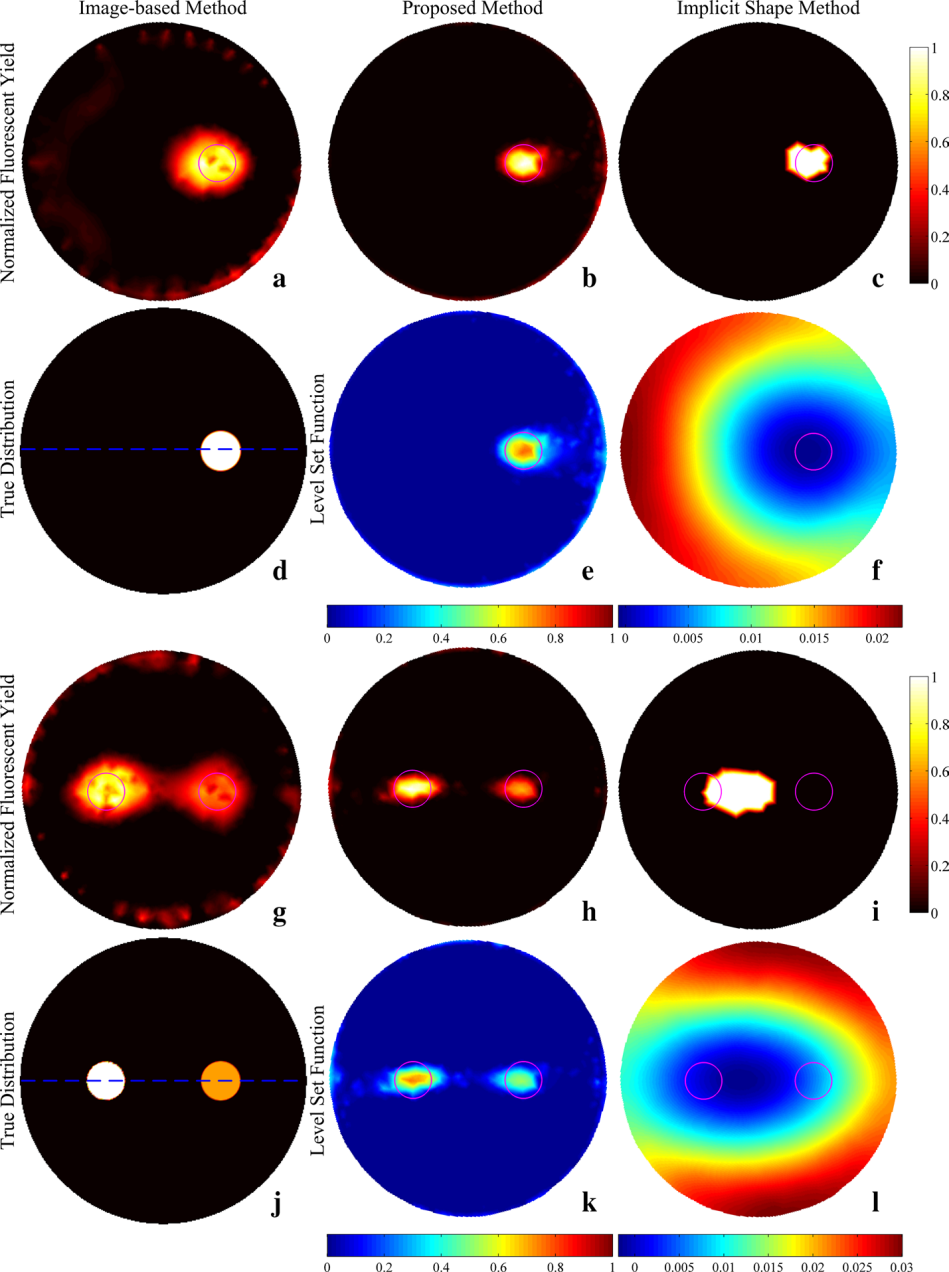
Reconstruction results of phantom studies. **a**–**c** Distributions of fluorescent yield reconstructed with the image-based method, the proposed method, and the implicit shape method for single target, respectively. **d** True distribution of fluorescent yield for single target. **e**, **f** Distributions of level set function reconstructed with the proposed method and the implicit shape method for single target, respectively. **g**–**l** Corresponding results for double targets. The reconstructions with the implicit shape method were implemented without the *priori* information about the number of targets

**Fig. 7:**
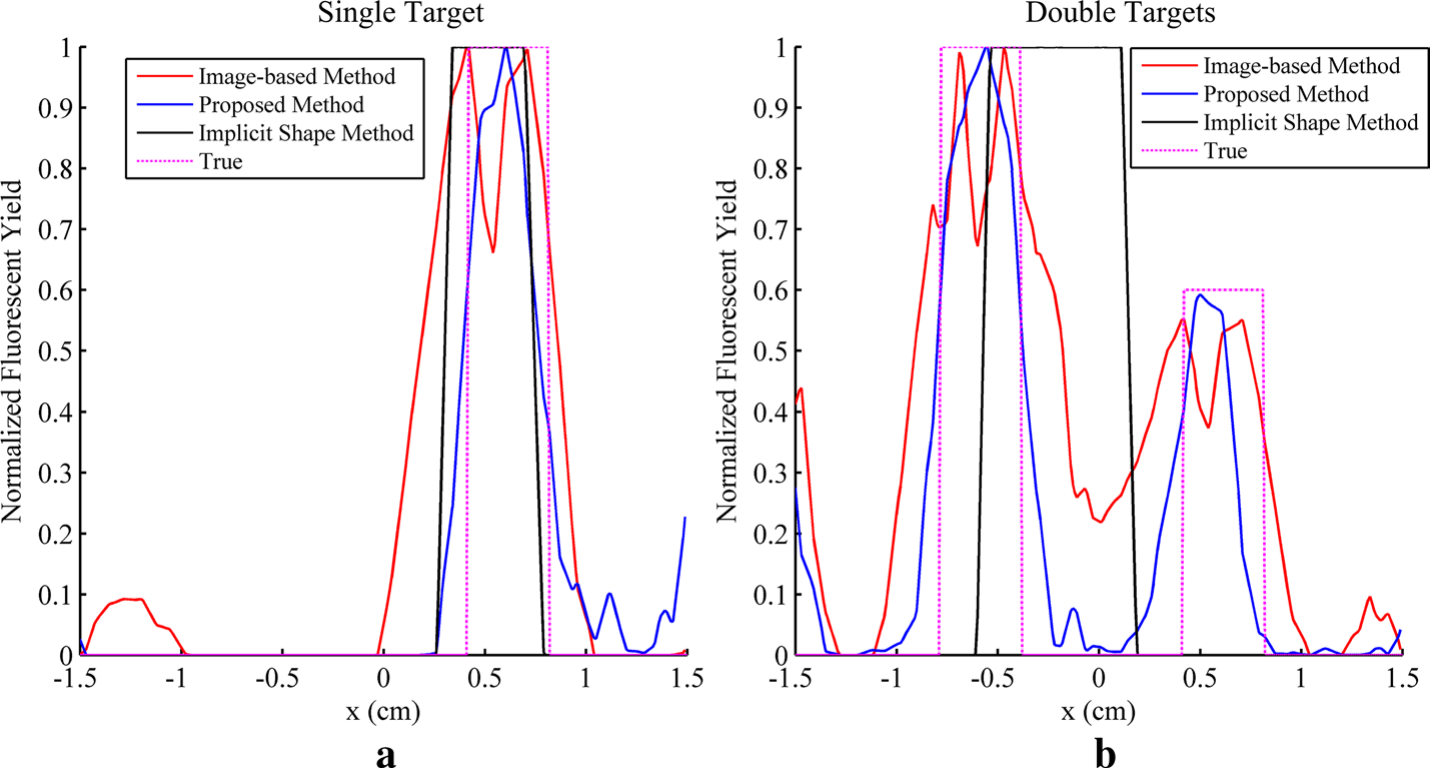
Profiles of normalized fluorescent yield along the *blue dotted lines* in Fig. 6d and j. **a** Result for Fig. 6d. **b** Result for Fig. 6j

**Fig. 8:**
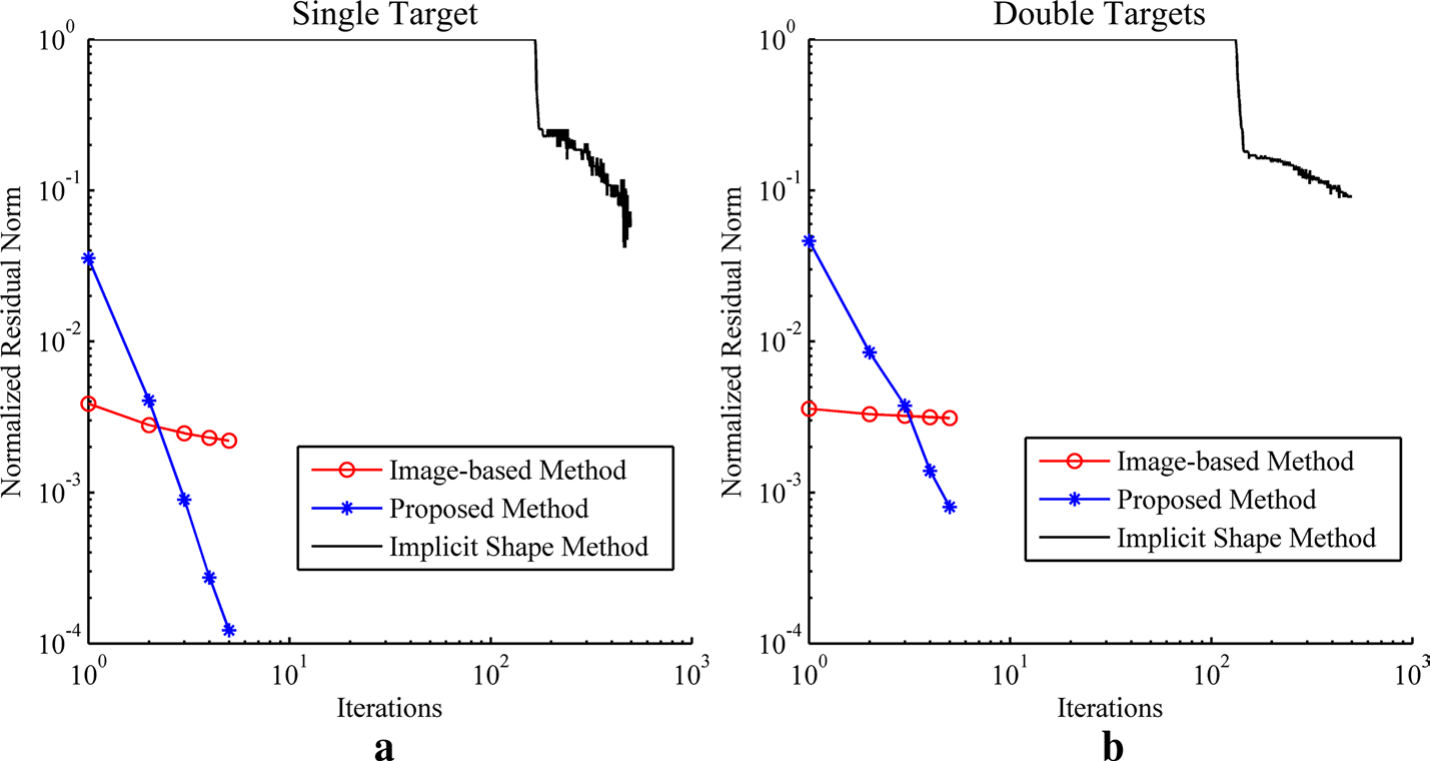
Residual norms as a function of iteration indices for phantom studies. **a** Result for single target. **b** Result for double targets

The reconstruction results of simulations and phantom experiments are shown in Figs. 3, 4, 5, 6, 7 and 8, respectively. Figures 3 and 6 show the distributions of fluorescent yield normalized with the maximum and the corresponding distributions of level set function. Figures 4 and 7 show the profiles of normalized fluorescent yield along the blue dotted lines in Figs. 3d, j and 6d, j. Figures 5 and 8 give the residual norms as a function of iteration indices. To compare the convergence speeds of different methods, the residual norms are also normalized with the maximum. To evaluate the reconstruction results quantitatively, the contrast to noise ratio (CNR) and Pearson correlation (PC) [35] were used, which are defined as follows:

23 $$CNR = \frac{{\frac{1}{T}\sum\nolimits_{i = 1}^{T} {(x_{i} - x_{back} )} }}{{\sqrt {\frac{{a_{tar} }}{T}\sum\nolimits_{i = 1}^{T} {\sigma_{i}^{2} } + \sigma_{back}^{2} a_{back} } }}$$

24 $$PC(X_{tru} ,X_{rec} ) = \frac{{COV(X_{tru} ,X_{rec} )}}{{\sigma (X_{tru} )\sigma (X_{rec} )}}$$

where *x*_*i*_ and *x*_*back*_ denote the mean value of fluorescent yield within the true region of the *i*th target and the background, respectively. σ_*i*_ and σ_*back*_ are the corresponding variances. *a*_*tar*_ and *a*_*back*_ represent the ratios of the areas, which are given as *a*_*tar*_ = *A*_*tar*_/*A* and *a*_*back*_ = *A*_*back*_/*A*, where *A*_*tar*_, *A*_*back*_, and *A* denote the area of targets, the area of background, and the total area, respectively. *T* is the number of targets. *X*_*true*_ and *X*_*rec*_ are the vectors of fluorescent yield for the true and reconstructed distribution, respectively. The *COV* denotes the covariance and σ is the standard deviation. Higher CNR indicates better differentiability between the targets and the background, i.e. better image quality. The metric PC is used to describe the similarity between the true distribution and the reconstructed one. The CNRs and PCs of the reconstruction results of the simulation and phantom studies are listed in Tables 1 and 2.

**Table 1:** CNRs and PCs of reconstruction results of simulation studies

	Single target		Double targets	
	CNR	PC	CNR	PC
Image-based method	6.2905	0.6341	4.4873	0.6533
Proposed method	17.969	0.9221	10.945	0.9027
Implicit shape method	12.135	0.8486	−0.0034	0.0052

**Table 2:** CNRs and PCs of reconstruction results of phantom studies

	Single target		Double targets	
	CNR	PC	CNR	PC
Image-based method	3.2547	0.6503	4.6587	0.6407
Proposed method	5.4847	0.8134	5.9463	0.7351
Implicit shape method	5.2447	0.7971	0.7426	0.1645

Figures 3a–f and 6a–f show that all of the three methods are capable of recovering the distribution of fluorescent yield for single target, but the results of image-based method are more blurry than the results of the other two methods due to the over-smoothness. Because of the Heaviside function used in the implicit shape method, the results of the implicit shape method show explicit boundaries, while the results of the proposed method show blurry boundaries due to the cosine function. However, the results of the implicit shape method indicate incapability of recovering boundaries exactly matching the true shapes due to the irregularity of the meshes and the ill-posedness of the inverse problem. As a result, the CNRs and PCs of the results of the proposed method are higher than those of the implicit shape method as shown in Tables 1 and 2. In general, the proposed method and the implicit shape method achieve higher image clarity than the image-based method and have similar performance for the reconstruction of single target. However, through Figs. 3g–l and 6g–l as well as the corresponding CNRs and PCs in Tables 1 and 2, it can be found that the implicit shape method is incapable of reconstructing the two targets without the *priori* information about the number of targets but the proposed method still works. The inability of the implicit shape method reconstructing double targets with different fluorescent yields derives from that the level set function is unable to represent multiple targets unless these targets have the same fluorescent yield. If multiple regions can be recognized during the iterative process, the coefficient *x*_*f*_ in Eq. (5) that describes the fluorescent yield of the targets can be split into multiple coefficients to represent multiple targets. Nevertheless, this condition is usually difficult to meet especially when the targets are close to each other like Figs. 3l and 6l. To avoid the overlap of the reconstructed shapes of multiple targets, multiple coefficients and the corresponding shapes should be initialized before the start of the iterative process, however, a good guess of the distribution of fluorescent yield and the number of targets is essential for the initialization. Alternatively, for multiple targets, multiple levels of a level set function or more than one level set function can be adopted, but both of them need to be initialized with the information about the number of targets.

As a variation of the Newton method, the LM method converges much faster than the gradient descent method which is a first order method as shown in Figs. 5 and 8. Five iterations are sufficient for the LM method while hundreds of iterations are required for the gradient descent method. Furthermore, the gradient descent method needs a number of iterations to make the level set function decline to minus and during these iterations the residual norm does not vary because there is no region restricted by the level set function. It leads to a flat section in the curve of residual norm versus iteration indices as shown in Figs. 5 and 8 and the length of the flat section are controlled by the initial conditions including the step length and the initial values of level set function and fluorescent yield. The initial conditions of the gradient descent method are more difficult to be determined than the LM method because the gradient descent method is usually unable to converge when the initial conditions are chosen improperly. The choice of the initial value of level set function and the choice of step length are contradicted with each other. When a large step length and a small initial value of level set function are used, the flat section can be shortened but the residual norm may increase along with the increasing of the iteration index, which results in divergence. On the contrary, a small step length and a large initial value of level set function lead to a low convergence speed, i.e. more iterations are required. Moreover, it is difficult to avoid the iterations those increase the residual norm in the gradient descent method, thus the curve of residual norm versus iteration indices commonly appears as a sawtooth pattern that the residual norm increases and decreases alternately along with the increase of iteration index, which can be observed in Figs. 5 and 8. A varied step length may solve the problem while how to change the step length is still intractable and the choice of the step length would be time-consuming. The difficulty of the choice of step length also derives from that the gradient used in the artificial time evolution approach is not the true gradient of the object function because the Dirac function, the derivative of the Heaviside function, is omitted in the gradient. Rigorously, the gradient is only proper for the positions with the level set function equal to 0. The Dirac function makes the reconstruction results so sensitive to the step length that the step length is difficult to be chosen.

**Fig. 9:**
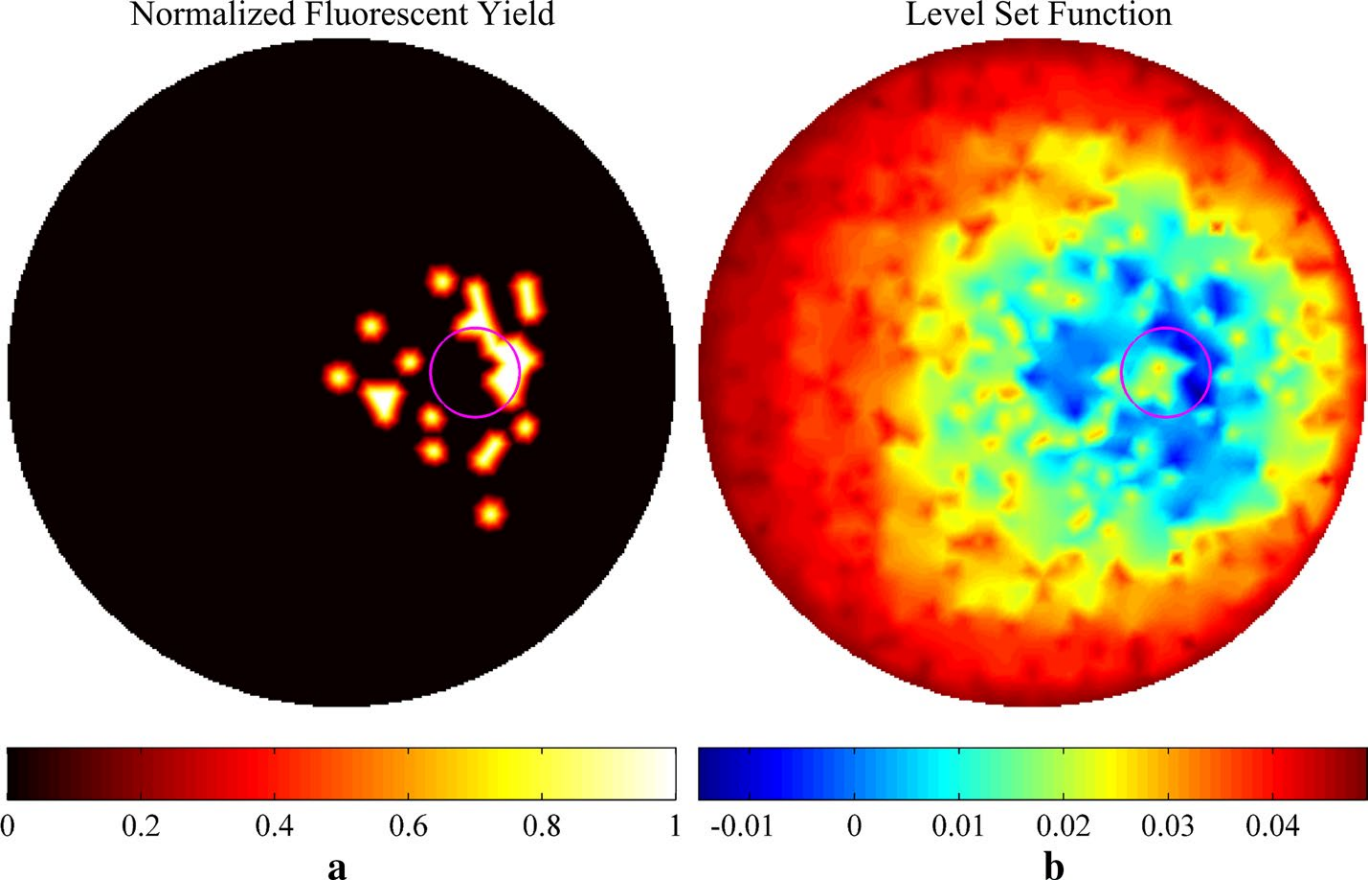
Reconstruction results of implicit shape method without low-pass filter for single target. **a** Distribution of fluorescent yield normalized with maximum. **b** Distribution of level set function

Figures 3a, g and 6a, g show that the reconstruction results of the image-based method are not homogeneous within the regions of targets and there are caves in the reconstructed targets. This phenomenon can also be observed in Figs. 4 and 7. It is caused by the irregularity of the meshes. The reconstructed values of fluorescent yield of nodes are affected by the sizes of the elements which contain these nodes. A reconstruction strategy with double levels of mesh that performs the forward calculations on a triangular or tetrahedral mesh and implements the reconstruction on a square or cubic mesh can solve this problem but will complicate the reconstruction process. As an alternative, a low-pass filter can be applied to smooth the reconstruction results [36]. In addition, the irregularity of the meshes also distorts the reconstruction results of the implicit shape method that it makes the regions restricted by the level set function are divided into pieces as shown in Fig. 9. To solve this problem, the results of the implicit shape method in Figs. 3, 6, 7 and 8 are obtained with a process smoothing the distributions of level set function through the low-pass filter after each iteration. In parallel, the proposed method is not influenced by the irregularity of the meshes as shown in Figs. 3b, e, h, k and 6b, e, h, k.

The difference between the proposed method and the implicit shape method derives from the replacement of the Heaviside function with the cosine function in Eq. (7). The primary defect of the Heaviside function is the nonderivability of its derivative the Dirac function. It results in the unavailability of second order methods. In parallel, the derivative of cosine function, sine function, is derivable. Consequently, the second order methods can be implemented. Moreover, the gradient of the object function for the Heaviside function includes the Dirac function which cannot be calculated numerically and has to be omitted in reconstruction. The omitted Dirac function in the gradient leads to that the reconstruction results are sensitive to the step length so that the step length is difficult to be determined. In comparison, the utilization of the cosine function avoids this problem. Finally, the Heaviside function fixes the fluorescent yield on two values *x*_*b*_ and *x*_*f*_, which results in the requirement of the *priori* information about the number of targets. On the contrary, the cosine function makes the fluorescent yield vary between *x*_*b*_ and *x*_*f*_, hence the *priori* information about the number of targets is not required any more. However, the variable fluorescent yield also results in the blurring of the reconstructed shapes. This is the disadvantage of the cosine function.

Generally, the proposed method can be considered as a compromise between the image-based method and the implicit shape method. Taking advantage of the Newton-type method, the image-based method is good at fast convergence and stable reconstruction but suffers from low image clarity. On the contrary, the implicit shape method provides high image clarity with the level set function but suffers from a slow convergence speed and unstable reconstruction due to the utilization of first order methods. The proposed method implements both the Newton-type method and the level set function to achieve the advantages of both the two methods. However, the proposed method is incapable of obtaining images with explicit boundaries because the cosine function blurs the shapes of the reconstruction results.

## Conclusions

In conclusion, a shape-based reconstruction scheme of FMT with cosinoidal level set method is proposed in this paper. This reconstruction method replaces the Heaviside function with a cosine function in the classical implicit shape method so as to take use of the Levenberg–Marquardt method. The proposed method provides a faster convergence speed than the implicit shape method and higher image clarity than the image-based reconstruction method. Furthermore, the proposed method does not need to know the number of targets and avoids the choice of step length, which is an intractable problem in the gradient descent method. As a result, the proposed method performs more stably than the implicit shape method.

## Abbreviations

FMT: fluorescence molecular tomography
FEM: finite element method
LM: Levenberg–Marquardt
ICG: indocyanine green
CNR: contrast to noise ratio
PC: Pearson correlation
